# Hypertension-Induced Renal Injury: From Pathophysiology to Therapeutic Perspectives

**DOI:** 10.3390/biomedicines14030595

**Published:** 2026-03-06

**Authors:** Ning Zhou, Su-Ye Zhong, Pan Gao, Fang-Fang He, Chun Zhang

**Affiliations:** Department of Nephrology, Union Hospital, Tongji Medical College, Huazhong University of Science and Technology, Wuhan 430022, China; nzhou2527@126.com (N.Z.); zhongsuye2000@163.com (S.-Y.Z.); panpangao1121@163.com (P.G.)

**Keywords:** hypertension, renal injury, molecular mechanisms, biomarkers, therapeutic strategies

## Abstract

Hypertension-induced renal injury is a major cause of chronic kidney disease and end-stage renal disease. Increasing evidence indicates that disease progression is not driven solely by hemodynamic stress but results from the interplay of multiple molecular mechanisms. In this review, we propose a stage-structured and network-based framework to systematically integrate current mechanistic insights into hypertension-induced renal injury. Early events, mainly including endothelial dysfunction and renal hypoxia, establish a permissive microenvironment for disease progression. These insults activate amplifying pathways such as the renin–angiotensin–aldosterone system (RAAS) overactivation, oxidative stress, immune and inflammatory responses, and sympathetic nervous system hyperactivity, which interact through cross-talk and positive feedback loops. Ultimately, these signals converge on fibrotic programs characterized by epithelial–mesenchymal transition (EMT), fibroblast activation, and extracellular matrix deposition, leading to irreversible structural remodeling and functional decline. Furthermore, epigenetics, the gut–kidney axis, autophagy dysfunction and renal aging also contribute to this process. We highlight two critical and underappreciated aspects: the existence of a permissive ‘early-window’ dominated by endothelial dysfunction and hypoxia, which sets the stage for later amplification; and the hierarchical interplay between amplifying mechanisms where cross talk creates self-reinforcing loops that may explain therapeutic resistance. In addition, this review highlights emerging biomarkers for early diagnosis and disease monitoring, and discusses therapeutic advances that extend beyond blood pressure control to disease-modifying interventions that confer renoprotective effects. By integrating molecular mechanisms with diagnostic and therapeutic perspectives, this review provides a comprehensive framework for early detection and precision intervention in hypertension-induced renal injury.

## 1. Introduction

Hypertension-induced renal injury is a major contributor to chronic kidney disease (CKD) worldwide and continues to pose a substantial public health burden [1,2]. Hypertension affects more than one billion individuals globally, with its prevalence rising steadily, particularly in low- and middle-income regions [3]. As one of the leading etiologies of CKD and end-stage renal disease (ESRD), hypertension-induced renal injury accounts for a significant proportion of kidney-associated morbidity and mortality, thereby exerting profound clinical and socioeconomic impacts [4,5].

The pathogenesis of hypertension-induced renal injury is complex and multifactorial. Sustained elevation of systemic blood pressure disrupts renal autoregulatory mechanisms, leading to glomerular hyperfiltration, vascular remodeling, endothelial dysfunction, oxidative stress, and progressive tubulointerstitial fibrosis. These processes ultimately converge to drive irreversible structural and functional deterioration of the kidney. Importantly, accumulating evidence indicates that renal injury in hypertension is not solely a consequence of hemodynamic stress but is critically mediated by non-hemodynamic mechanisms, including neurohumoral activation, immune and inflammatory responses, metabolic dysregulation, and maladaptive cellular remodeling [6,7,8].

Despite substantial advances in antihypertensive therapy, particularly the widespread use of RAAS inhibitors and other blood pressure-lowering agents, a significant proportion of patients continue to experience progressive renal impairment [9].This observation highlights the limitations of blood pressure-centered management strategies and emphasizes the need for a more comprehensive understanding of the molecular and cellular pathways driving hypertensive renal injury. In recent years, emerging data have increasingly supported a network-based view of disease pathogenesis, in which interconnected signaling pathways—rather than isolated molecular events—collectively govern renal injury progression.

Although numerous reviews have addressed individual pathogenic mechanisms or therapeutic approaches in hypertension-induced renal injury, an integrated framework that links molecular pathogenesis with diagnostic, monitoring, and mechanism-based therapeutic strategies remains insufficiently developed. In this review, we provide a comprehensive and network-oriented overview of the molecular and cellular mechanisms underlying hypertension-induced renal injury from early initiating stage, amplifying stage, effect stage and some regulatory pathways with particular emphasis on the crosstalk among hemodynamic stress, RAAS overactivation, oxidative stress, inflammatory-immune signaling, fibrotic remodeling, and key regulatory mechanisms. Furthermore, we synthesize recent advances in diagnostic and monitoring strategies and discuss established and emerging therapeutic approaches that extend beyond blood pressure control. By bridging mechanistic insights with clinical translation, this review aims to offer an integrated framework to support more precise diagnosis, dynamic monitoring, and mechanism-based management of hypertension-induced renal injury.

## 2. Pathophysiology of Hypertension-Induced Renal Injury

Hypertensive renal injury involves multilayered structural and functional alterations across the renal microvasculature, glomeruli, and tubulointerstitium. Persistent hypertension disrupts the kidney’s autoregulatory capacity, initiating a cascade of pathological responses. The characteristic lesions include microvascular remodeling, glomerulosclerosis, and tubulointerstitial fibrosis. Although these abnormalities appear to arise in distinct anatomical compartments, they interact extensively throughout disease progression, reinforcing one another and driving a self-perpetuating cycle of renal injury.

### 2.1. Hypertension-Induced Renal Injury in Small Vessels

Renal microvascular remodeling is one of the earliest and most influential events in hypertension-induced renal injury. Under physiological aging, both afferent and efferent arterioles undergo proportional medial thickening. In contrast, chronic hypertension induces disproportionate medial smooth muscle hypertrophy, intimal fibrosis, and luminal narrowing, resulting in a marked increase in the wall-to-lumen ratio [9]. Hyaline deposition further compromises vascular integrity, with afferent arterioles more severely affected due to higher exposure to systemic pressure waves [10].

As vascular stiffness progresses, renal autoregulatory capacity declines, exposing glomerular capillaries to excessive pressure and reducing cortical perfusion, which contributes to hypoxia. These vascular changes propagate downstream, promoting glomerular injury and tubulointerstitial hypoxia, making microvascular disease a central upstream driver of subsequent renal pathology.

### 2.2. Hypertension-Induced Renal Injury in Glomerulus

Due to microvascular dysregulation and hemodynamic disturbance, the glomerulus becomes directly exposed to abnormally elevated pressure. In the early stage, the kidney maintains filtration through efferent arteriolar constriction [11]. However, prolonged transmission of systemic pressure eventually leads to endothelial injury, increased glomerular basement membrane permeability, and protein leakage [12]. Mechanical stress and inflammatory signaling stimulate mesangial cell proliferation and enhance extracellular matrix (ECM) production, resulting in mesangial expansion and basement membrane thickening, ultimately progressing to glomerulosclerosis [10,13].

**Figure 1:**
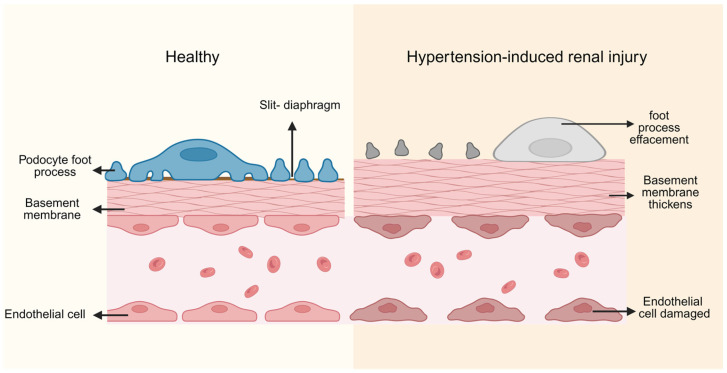
The glomerular filtration barrier in progression of hypertension-induced renal injury. The glomerular filtration barrier includes specialized endothelial cells, basement membranes and podocyte slit diaphragms. When injury occurs, endothelial cells are damaged, the basement membrane thickens and the podocytes foot processes undergo effacement and detach from the basement membrane. Created in BioRender. Zhou, N. (2026) https://BioRender.com/q366s6e (accessed on 29 January 2026).

Podocytes, essential components of the glomerular filtration barrier, normally interdigitate their foot processes along the glomerular basement membrane, forming slit diaphragms that are crucial for maintaining selective filtration. Under sustained hypertensive stress, podocytes develop cytoskeletal disorganization, foot process effacement, slit diaphragm disruption, and detachment (Figure 1) [14,15,16]. Given their extremely limited regenerative capacity, podocyte loss becomes a critical turning point that accelerates glomerulosclerosis.

There is pronounced cross-talk between glomerular injury and the tubulointerstitial compartment. Proteinuria increases the metabolic and inflammatory burden on the proximal tubules, while factors released from injured podocytes activate interstitial fibroblasts. Consequently, tubulointerstitial fibrosis acts as a downstream amplifier of glomerular injury.

### 2.3. Hypertension-Induced Renal Injury in Tubular and Interstitial

Tubular and interstitial alterations induced by hypertension are characterized primarily by tubular atrophy and the excessive accumulation of ECM within the tubulointerstitium [17,18]. Under sustained mechanical stress and hypoxic conditions, tubular epithelial cells undergo metabolic reprogramming characterized by impaired fatty acid oxidation, which subsequently triggers EMT. This process results in the loss of epithelial polarity and intercellular junctions, acquisition of mesenchymal-like properties, and abnormal patterns of cell migration and proliferation.

During hypertension, angiotensin II (Ang II) promotes the recruitment of inflammatory cells into the kidney by upregulating adhesion molecules, cytokines, and chemokines, while simultaneously activating the NF-κB pathway through AT1 receptor signaling, thereby enhancing the expression of pro-inflammatory genes [19]. AT2 receptors also contribute to inflammatory cell recruitment, further amplifying local inflammation. Therefore, combined inhibition of AT1 and AT2 receptor activity, along with blockade of the NF-κB pathway, may effectively attenuate hypertension-induced tubular–interstitial inflammation and fibrosis

## 3. Molecular Mechanism of Hypertension-Induced Renal Injury

Hypertension-induced renal injury results from a coordinated network of molecular disturbances triggered by chronic blood pressure elevation. Chronic elevation of systemic blood pressure imposes sustained mechanical and hemodynamic stress on the renal microcirculation, which initiates early injury characterized by endothelial dysfunction, impaired autoregulation, and microvascular rarefaction. These vascular alterations reduce perfusion efficiency and predispose the kidney to tissue hypoxia and metabolic stress, creating a permissive environment that facilitates downstream pathogenic activation.

Subsequently, multiple amplifying mechanisms are engaged, including RAAS overactivation, oxidative stress, inflammatory cytokine production, immune cell recruitment, and sympathetic nervous system (SNS) overactivity. These pathways interact extensively: Ang II promotes ROS production, ROS enhances NF-κB signaling, and inflammatory mediators further activate RAAS and SNS, forming self-reinforcing loops that propagate injury through glomerular, tubular, and interstitial compartments.

The sustained activation of these upstream and amplifying signals culminates in effector mechanisms, notably EMT, fibroblast activation, and excessive ECM accumulation, which drive irreversible tubulointerstitial fibrosis.

Disease susceptibility and progression are further modulated by epigenetic regulation, gut–kidney metabolic interactions, and autophagy dysfunction, which integrate environmental and cellular stress cues.

**Figure 2:**
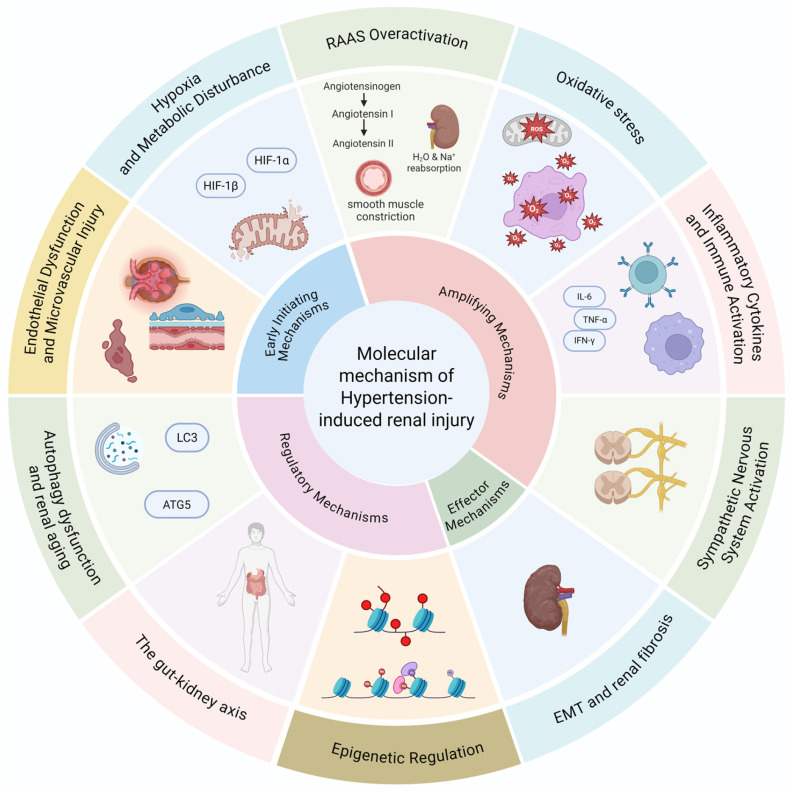
Integrated molecular network of hypertension-induced renal injury. Hypertension-induced renal injury is driven by an interconnected molecular network. At the initiating level, hemodynamic stress initiates endothelial dysfunction and microvascular injury, leading to renal hypoxia and metabolic disturbance. At the amplifying level, RAAS overactivation, oxidative stress, immune–inflammatory signaling, and sympathetic activation interact to reinforce renal injury. Ultimately, at the effector level, these events converge on epithelial–mesenchymal transition and renal fibrosis. Disease progression is further modulated by regulatory mechanisms, including epigenetic regulation, gut–kidney axis dysregulation, impaired autophagy and renal aging. Created in BioRender. Zhang, C. (2026) https://BioRender.com/n5px93b (accessed on 11 January 2026).

Overall, hypertension-induced renal injury represents a multi-level pathogenic network rather than a linear sequence. Understanding these interconnected mechanisms provides the foundation for developing targeted therapeutic strategies discussed in subsequent sections. To better integrate the interconnected mechanisms described above, we propose an integrated network model summarizing the dynamic progression of hypertension-induced renal injury (Figure 2).

### 3.1. Early Initiating Mechanisms

#### 3.1.1. Endothelial Dysfunction and Microvascular Injury

Endothelial cells constitute the inner lining of renal microvessels and act as dynamic mechanosensors that detect alterations in hemodynamic forces [20]. By releasing a wide spectrum of vasodilatory and vasoconstrictive mediators, they play a central role in maintaining vascular tone and renal microcirculatory homeostasis [21]. Among these mediators, nitric oxide (NO), generated by endothelial nitric oxide synthase (eNOS), is essential for vasodilation and exerts anti-inflammatory, anti-thrombotic, anti-proliferative, and antioxidative functions [22]. Under hypertensive conditions, eNOS activity is impaired and NO is rapidly degraded by reactive oxygen species, leading to decreased NO bioavailability [23]. This reduction promotes vasoconstriction, increases renal vascular resistance, and ultimately contributes to insufficient renal perfusion [24].

Sustained hypertension imposes excessive shear stress on glomerular and preglomerular vessels, disrupting endothelial integrity and increasing permeability to macromolecules [25]. These structural abnormalities accelerate glomerular hyperfiltration and compromise the filtration barrier, thereby initiating maladaptive downstream responses. In addition, microvascular rarefaction decreases renal perfusion capacity and aggravates local hypoxia, creating an environment that favors oxidative and inflammatory activation.

Mechanical stress also induces marked alterations in endothelial cytoskeletal architecture, impairing mechanotransduction essential for vascular responsiveness. Increased matrix metalloproteinase activity and disturbances in integrin–extracellular matrix interactions weaken the myogenic response of afferent arterioles [26]. As autoregulation deteriorates, the kidney loses its ability to buffer systemic blood pressure fluctuations, allowing systemic hypertension to be directly transmitted to glomerular capillaries, thereby accelerating endothelial and microvascular injury and promoting the progression of hypertension-induced renal injury [27].

Taken together, endothelial dysfunction constitutes a pivotal initiating event in hypertension-induced renal injury. Reduced NO bioavailability, microvascular structural alterations, and impaired autoregulation converge to create a hypoxic, pro-oxidative, and pro-inflammatory microenvironment, which predisposes the kidney to subsequent RAAS activation, oxidative stress, and immune-mediated injury. In support of the regulatory potential of NO–eNOS signaling, recent studies have shown that the herbal pair SYR-NX attenuates hypertensive renal injury by enhancing eNOS expression and improving endothelial metabolic homeostasis [28], while dietary interventions such as Coriandrum sativum have been reported to increase NO levels and ameliorate endothelial dysfunction in hypertension models [29]. These findings highlight the therapeutic relevance of restoring eNOS-dependent endothelial protection in the early stages of hypertensive renal injury.

#### 3.1.2. Renal Hypoxia and Metabolic Disturbance

Hypoxia is a central pathogenic driver in hypertension-induced renal injury, producing a redox imbalance that increases intracellular reactive oxygen species (ROS) production while relatively diminishing antioxidant defenses [30]. Elevated ROS under hypoxic conditions damages lipids, proteins and nucleic acids, disrupts membrane integrity, and impairs organelle function, notably mitochondrial membranes, thereby perturbing cellular energy metabolism and amplifying oxidative injury [31]. In renal parenchyma, hypoxia also stimulates excessive synthesis and deposition of extracellular matrix components such as collagen and fibronectin by mesangial cells and podocytes, contributing to glomerulosclerosis and loss of filtration function [32]. Hypoxia-induced structural injury and ECM accumulation have also been demonstrated in experimental renal hypoxia models, further confirming its crucial role in renal structural remodeling [33].

At the molecular level, hypoxia stabilizes hypoxia-inducible factors (HIFs), the master transcriptional regulators of the hypoxic program [34]. HIF accumulation triggers a broad transcriptomic response that modulates metabolism, angiogenesis and profibrotic signaling. Notably, angiotensin II can induce endothelial HIF-1α expression via NF-κB (p65/RelA)-dependent mechanisms, which in turn upregulates TGF-β, promotes extracellular matrix synthesis, and accelerates renal fibrosis [35]. Furthermore, hypoxia-induced HIF activation also modulates inflammatory and angiogenic pathways, reinforcing tissue remodeling and metabolic adaptation under chronic hypoxic stress [36]. However, the net effect of HIF activation in chronic kidney disease is context-dependent: genetic HIF stabilization often favors pro-fibrotic outcomes, whereas pharmacological HIF activation in some animal models can be nephroprotective; the timing, duration, HIF subtype and cell-specific responses critically determine the balance between repair and fibrosis [37].

Hypoxia also precipitates cell death pathways that contribute to structural loss. Low oxygen tension activates pro-apoptotic mediators, such as Bax and suppresses anti-apoptotic factors, such as Bcl-2, leading to mitochondrial permeability transition, cytochrome c release, caspase activation and apoptosis; in severe or prolonged hypoxia, necrosis may ensue due to catastrophic metabolic failure [38].

Collectively, hypoxia-driven oxidative stress, maladaptive metabolic reprogramming and cell death establish a feed-forward milieu that promotes inflammation, extracellular matrix accumulation and progressive renal dysfunction.

### 3.2. Amplifying Mechanisms

#### 3.2.1. RAAS Overactivation

The renin–angiotensin–aldosterone system (RAAS) acts as a central pathogenic axis in hypertension-induced renal injury, with excessive angiotensin II (Ang II) generation serving as its key effector [39]. Ang II activates AT1 receptors widely expressed in renal vascular, glomerular, and tubular cells, initiating Ca^2+^ dysregulation and triggering a broad range of downstream pathogenic pathways [40,41]. A major hemodynamic effect of Ang II is preferential constriction of the efferent arteriole, sharply elevating intraglomerular pressure and causing early hyperfiltration; chronic transmission of this high-pressure load subsequently induces glomerulosclerosis, basement membrane thickening, podocyte depletion, and progressive filtration decline [42,43].

Beyond hemodynamic injury, Ang II enhances extracellular matrix deposition by upregulating pro-fibrotic mediators such as TGF-β and CTGF [44]. It can further activate AT1-dependent Smad pathways independently of TGF-β, whereas Smad7 functions as an inhibitory checkpoint that limits excessive fibrogenesis [45]. Importantly, recent findings show that ciliary neurotrophic factor (CNTF) modulates Ang II-induced pressor responses through JAK2/STAT3 signaling, and CNTF deficiency significantly attenuates hypertension-associated renal injury [46], highlighting an additional regulatory layer within the Ang II–AT1R axis. Another study found Dectin-1 expression on CD68 + macrophages was significantly elevated in the kidney after Ang II infusion. Ang II-induced renal dysfunction, interstitial fibrosis, and immune activation were significantly attenuated in Dectin-1-deficient mice. Specifically, macrophage-derived Dectin-1 activated neutrophil migration into the kidney and upregulated TGF-β1 secretion, leading to renal dysfunction and fibrosis in response to Ang II infusion [47].

Aldosterone represents another critical RAAS effector contributing substantially to hypertensive renal injury. Sustained aldosterone elevation enhances mineralocorticoid receptor (MR) activation in tubular epithelial and vascular cells, promoting sodium retention, oxidative stress, pro-inflammatory signaling, and fibroblast activation [48]. Persistent MR activation drives extensive interstitial inflammation and fibrosis, thereby coupling hemodynamic injury with chronic parenchymal remodeling. Although MR antagonists (MRAs) are recommended in therapy, their use is limited by risks such as hyperkalemia and incomplete inhibition of nongenomic MR effects [49]. Aldosterone synthase inhibitors (ASIs) have emerged as promising alternatives, with phase II trials demonstrating reductions in resistant hypertension and albuminuria [50]. Given their complementary mechanisms, combination therapy with MRAs and ASIs may provide a more comprehensive blockade of aldosterone-driven pathways and represents a feasible future strategy for improving outcomes in hypertension-induced renal injury.

#### 3.2.2. Oxidative Stress

Mitochondria are semi-autonomous organelles responsible for diverse metabolic activities, including anabolic processes and ATP synthesis via oxidative phosphorylation [51]. Under hypertensive conditions, mitochondrial dysfunction becomes the predominant source of oxidative stress, leading to excessive production of reactive oxygen and nitrogen species (ROS/RNS) that disrupt renal cellular homeostasis and initiate kidney injury [52,53]. A key contributor to this process is pathological mitochondrial fragmentation mediated by the ROCK1–Drp1 pathway; inhibition of ROCK1 by hydroxyfasudil suppresses phosphorylated Drp1-dependent fission, attenuates mitochondrial ROS accumulation, and alleviates CKD-associated tissue damage [54].

The excessive ROS generated from dysfunctional mitochondria promotes ferroptosis, an iron-dependent form of regulated cell death. Upregulation of HDAC3 suppresses GPX4 expression, weakens antioxidant defense, and drives tubular epithelial ferroptosis, facilitating AKI-to-CKD transition [55]. Ferroptosis also contributes to endothelial cell injury, linking oxidative stress with microvascular dysfunction in hypertension-induced renal damage [56]. Together, these findings position ferroptosis as a key downstream effector of oxidative stress-mediated renal injury.

Multiple endogenous pathways modulate oxidative stress severity in hypertension-induced renal injury. Activin A amplifies vascular oxidative stress, enhances vasoconstriction, and impairs endothelial-dependent relaxation, while its natural antagonist follistatin suppresses oxidative stress and improves vascular structure and function in hypertension models [57]. Additionally, Vascular Endothelial Growth Factor Receptor 3 (VEGFR3) expression is upregulated in renal proximal tubular cells under hypoxic or oxidative conditions. VEGFR3 exerts renoprotective effects by enhancing PARKIN-dependent mitophagy through regulating HSPA1L crotonylation at the K130 site, thereby mitigating Ang II-induced oxidative damage and preserving mitochondrial quality [58]. These pathways highlight the intricate regulatory network that determines the renal response to oxidative stress.

Beyond intrinsic cellular sources, oxidative stress is further amplified by immune-mediated mechanisms. In high-salt hypertension, infiltrating macrophages exhibit markedly elevated CD14 expression, which activates NADPH oxidase 2 (NOX2) and increases superoxide generation, exacerbating renal injury [59]. Notably, NOX2-derived superoxide is significantly reduced in cultured SSCD14^−^/^−^ rats, suggesting that targeting CD14 may serve as a promising therapeutic approach to attenuate oxidative stress-driven renal damage [59]. This immune amplification loop integrates oxidative stress with inflammatory activation in hypertension-induced renal injury.

#### 3.2.3. Inflammatory Cytokines and Immune Activation

Inflammatory cytokines and activated immune cells constitute a central pathogenic axis in hypertension-induced renal injury, bridging hemodynamic stress with progressive structural damage [60,61]. Persistent elevation of pro-inflammatory mediators not only disrupts endothelial and tubular homeostasis but also initiates chemokine-driven leukocyte recruitment, thereby transforming transient hemodynamic perturbations into sustained immune activation. As innate and adaptive immune responses intensify, macrophages, dendritic cells, T cells and B cells interact through multilayered cytokine networks to amplify renal inflammation and accelerate interstitial fibrosis [62]. Therefore, cytokine–immune activation represents a pivotal mechanistic hub linking early vascular injury to chronic renal remodeling and provides an essential foundation for understanding downstream effector pathways.

1. Inflammatory Cytokines

Inflammation is a central pathogenic axis in hypertension-induced renal injury, and the sustained elevation of pro-inflammatory cytokines represents the earliest driver transforming hemodynamic stress into immunologically mediated tissue damage [63]. Under chronic hypertension, endothelial dysfunction, tubular mechanical stress, and altered renal microcirculation collaboratively stimulate the production of inflammatory mediators within both intrinsic renal cells and infiltrating leukocytes.

TNF-α is one of the earliest and most influential cytokines involved in this process. Hypertension promotes the release of TNF-α from tubular epithelial cells, infiltrating macrophages, and resident immune cells, causing NF-κB activation, upregulation of ICAM-1/VCAM-1, increased endothelial permeability, and tubular apoptosis [64,65]. Experimental evidence demonstrates that TNF-α inhibition markedly reduces renal inflammation and tissue injury in angiotensin II-induced hypertension, whereas in the DOCA-salt model, TNF-α blockade does not lessen blood pressure and may even worsen hypertension due to distinct immune-hemodynamic contexts [66]. These findings establish TNF-α as a context-dependent regulator whose pathological significance differs across hypertensive models.

IL-6 further bridges hemodynamic stress with innate and adaptive immune responses. Via classical and trans-signaling, IL-6 engages JAK/STAT pathways, enhances CRP synthesis, promotes endothelial activation, and facilitates T cell and B cell expansion, collectively increasing renal susceptibility to immune infiltration [67]. IL-6 also synergizes with IL-1β to amplify cytokine cascades, thereby stabilizing a self-reinforcing inflammatory microenvironment within the hypertensive kidney. IL-17, predominantly derived from Th17 cells, acts as a potent amplifier of renal inflammation. IL-17 promotes chemokine production, facilitates leukocyte recruitment, and aggravates endothelial dysfunction [68,69]. Neutralization of IL-17A reduces blood pressure, renal inflammation, and structural injury in multiple hypertensive models, confirming its critical role in linking adaptive immune activation with renal tissue remodeling [70].

IFN-γ serves as a key mediator linking immune activation to renal sodium handling and inflammatory amplification. It upregulates angiotensinogen (AGT) expression in hepatocytes and proximal tubular epithelial cells, thereby enhancing local RAAS activity, promoting sodium retention, and aggravating hypertension-induced renal injury [71]. In the distal nephron, activated CD8^+^ T cells utilize an IFN-γ–PDL1 signaling pathway to increase sodium reabsorption; genetic deletion of IFN-γ or tubular PDL1 knockdown reduces T-cell homing to the kidney and attenuates hypertension in experimental models, demonstrating a causal immune–epithelial axis in blood-pressure regulation [72].Despite these pathogenic effects, the influence of IFN-γ is context dependent, with its functional outcomes varying according to dose, exposure duration, and immune milieu [73].

Collectively, these pro-inflammatory cytokines serve as upstream molecular switches that initiate and stabilize renal immune activation. Through the combined actions of TNF-α, IL-6, IL-17, and IFN-γ, the hypertension-induced renal injury transitions from a state of mechanical load to a sustained immunopathological process, laying the foundation for chemokine gradients, innate immune recruitment, and adaptive immune expansion.

2. Chemokines

Chemokines constitute a family of small 8–12 kDa proteins characterized by one to three disulfide bonds. Based on the number and arrangement of N-terminal cysteine residues that participate in these bonds, they are classified into four major subfamilies: CXC, CC, XC, and CX3C [74]. Among these, CCL2 and CCL5 have long been implicated in hypertension, yet emerging evidence demonstrates that their actions on blood pressure regulation and tissue injury are highly context-dependent. Beyond the CC family, several CXC chemokines also contribute to hypertension-induced renal injury. Notably, elevated circulating CXCL12 levels have been associated with cardiovascular risk factors, established cardiovascular disease, and increased hazards of incident myocardial infarction and all-cause mortality, suggesting a broader chemokine-mediated axis linking inflammation with target-organ damage [75]. Collectively, these chemokines orchestrate the recruitment of monocytes, macrophages, and lymphocytes into the hypertensive kidney, thereby sustaining local immune activation and amplifying renal injury.

3. Innate Immunity

Innate immunity plays a pivotal role in hypertension-induced renal injury, with macrophages and dendritic cells (DCs) serving as key effector populations. Hypertensive stimuli induce substantial macrophage infiltration into the kidney and promote phenotypic shifts characterized by increased M1 macrophages, which release pro-inflammatory mediators such as TNF-α and IL-6, whereas the anti-inflammatory and reparative functions of M2 macrophages become relatively diminished [76]. However, the mechanisms of macrophage polarization and functional transitions at different stages of renal injury are not well understood, and there are some differences in the identification and functional assessment of M1 and M2 type macrophages in different studies, which affects the accurate judgment of their roles in hypertensive renal injury [77].

DCs likewise exhibit heightened activation during hypertension [78]. Ang II stimulates NADPH oxidase within DCs, promoting neoantigen formation and augmenting T-cell responses, thereby contributing to elevated blood pressure and progressive renal injury [79]. Hypertension further alters the antigen-processing capacity of DCs, leading to increased production of pro-inflammatory cytokines [80]. In the 2K1C renovascular hypertension model, depletion of plasmacytoid DCs effectively lowered blood pressure, reduced inflammation and oxidative stress, and improved microvascular endothelial function, underscoring their pathogenic significance [81].

4. Adaptive Immunity

Within the adaptive immune landscape of hypertension-induced renal injury, T cells represent a central pathogenic driver. Hypertensive mechanical and oxidative stresses activate renal antigen-presenting cells, which stimulate T-cell activation and promote sustained T-cell infiltration into the kidney. CD4^+^ T cells, in particular, exacerbate renal inflammation through NOX2-dependent oxidative stress, thereby intensifying salt sensitivity and blood pressure elevation [82,83]. This redox imbalance fosters endothelial dysfunction, sodium retention, and vasoconstriction, all of which predispose the kidney to injury. In metabolic hypertension, T-cell deficiency in mice significantly attenuates high-fat-diet-induced blood pressure elevation and reduces renal sodium transporter expression, highlighting T cells as a critical link between metabolic stress and hypertensive responses [84].

Among T-cell subsets, Th17 cells and their effector cytokine IL-17 phosphorylate eNOS in vascular smooth muscle cells and outer membrane fibroblasts, decreasing NO production and leading to impaired vasodilatory function, and also promotes inflammatory cell recruitment and collagen synthesis, exacerbating renal fibrosis [85]. Cytotoxic T lymphocytes (CTLs) can kill target cells via the FASL/FAS pathway, leading to tubular epithelial cell loss and interstitial fibrosis [86]. The decrease in the number of regulatory T cells (Tregs) will weaken the inhibitory effect on inflammation, fail to inhibit macrophage activation and reduce the secretion of pro-inflammatory cytokines, which will aggravate renal injury [87]. A recent research found LGMN (legumain) deficiency in T cells prevents hypertension-induced renal injury by promoting Treg differentiation and function. Specifically targeting LGMN in Tregs may be an innovative approach for hypertension treatment [88].

Although current studies on the role of B cells in hypertension-induced renal injury remain limited, existing evidence indicates that B cells can exert distinct and potent pathogenic effects through multiple antibody-dependent mechanisms [89]. Hypertensive inflammation and oxidative stress promote B-cell activation and the production of autoantibodies. In specific metabolic contexts, such as hyperhomocysteinemia-associated hypertension, B-cell-derived anti-β2-GPI IgG deposits within glomeruli and induces ferroptosis, thereby worsening glomerulosclerosis and renal dysfunction [90].

Beyond this direct mechanism, insights from autoimmune kidney diseases suggest that immune complexes formed by autoantibodies and renal antigens may further activate the classical or lectin complement pathways, leading to membrane attack complex (MAC) formation and causing podocyte injury and disruption of the glomerular filtration barrier. In parallel, the Fc portion of these immune complexes can engage Fcγ receptors on macrophages and other effector cells, triggering phagocytosis, cytotoxic responses, and pro-inflammatory cytokine release, which amplify local renal inflammation [91]. In addition to serving as producers of pathogenic antibodies, B cells may also contribute through their function as antigen-presenting cells (APCs) [92].

Together, these mechanisms constitute a potential “B–T collaborative amplification loop,” in which antibody–complement–FcγR pathways enhance tissue-level inflammation and injury, while B-cell antigen presentation drives sustained T-cell activation; the two processes reinforce one another and may contribute to the progression and persistence of hypertensive renal injury.

There is a complex interaction between immune cells and inflammatory factors [93]. Cytokines produced by T cells activate macrophages, causing them to release more inflammatory factors and exacerbate the inflammatory response. Inflammatory factors secreted by macrophages promote the activation and proliferation of T cells, forming a vicious circle [94,95]. Dendritic cells present antigens to activate T cells and cytokines secreted by T cells affect the function of dendritic cells and regulate the immune response. This interaction is further enhanced after kidney injury occurs, leading to uncontrolled inflammatory response and increasing damage to the kidney, which in turn further aggravates hypertension by affecting the endocrine and metabolic functions of the kidney, such as increased renin secretion and decreased erythropoietin [96,97]. However, such interactions may vary in different individuals and disease stages, and the specific signaling pathways and regulatory mechanisms involved are not yet fully defined, with variations in the pattern and intensity of interactions observed in different studies, which poses a challenge to a deeper understanding of the roles of immunity and inflammation in renal injury in hypertension [98].

#### 3.2.4. Sympathetic Nervous System Activation

The sympathetic nervous system (SNS) is a major amplifying mechanism in hypertension-induced renal injury. Renal sympathetic efferent nerves regulate renal vascular tone, sodium handling, and renin secretion, profoundly influencing blood pressure and renal perfusion [99]. Persistent sympathetic overactivity induces renal vasoconstriction, elevates glomerular hydrostatic pressure, and enhances proximal tubular sodium reabsorption, thereby exacerbating volume-dependent hypertension and nephron mechanical stress [100]. In addition, a reinforcing positive feedback loop exists between the SNS and the RAAS, in which sympathetic excitation promotes renin release while angiotensin II further augments sympathetic drive [100].

Recent studies have identified a maladaptive kidney–brain–kidney neural circuit wherein renal afferent activation enhances central sympathetic outflow, driving progressive renal and cardiac injury; interruption of this circuit or renal denervation (RDN) ameliorates organ damage [101]. At the molecular level, sympathetic hyperactivity activates NADPH oxidase (NOX), increases ROS production, and promotes tubular injury and interstitial fibrosis, amplifying hypertensive renal damage [102].

The SNS also exerts powerful immunomodulatory effects. Norepinephrine enhances macrophage-derived cytokine production, such as TNF-α and IL-6, increases dendritic cell antigen presentation, and facilitates T-cell recruitment into the kidney, thereby escalating intrarenal inflammation [103]. Additionally, SNS activation promotes renal inflammation via the JAK–STAT pathway, whereas RDN attenuates this response in experimental models [104]. These findings underscore sympathetic hyperactivity as a central amplifying driver of hypertension-induced renal damage and support the SNS as a promising therapeutic target.

### 3.3. Effector Mechanisms

#### Epithelial–Mesenchymal Transition and Renal Fibrosis

Epithelial–mesenchymal transition (EMT) represents a central pathological program driving renal fibrogenesis. Sustained hypertensive injury, including mechanical stress, hypoxia, inflammation, and RAAS overactivation, primes tubular epithelial cells for EMT, enabling progression from reversible damage toward irreversible fibrosis [105].

**Figure 3:**
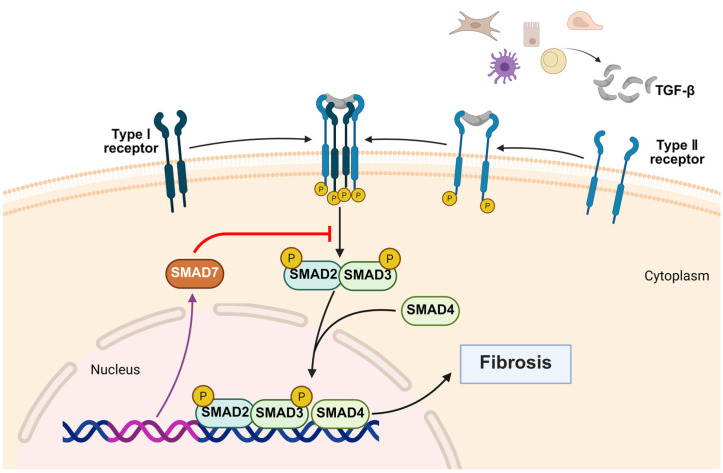
TGF-β/Smads signaling pathway in renal fibrosis. The TGF-β/Smads pathway plays a leading role in promoting renal fibrosis. When hypertension occurs, the injury stimulation causes various cells to secrete TGF-β. TGF-β binds to the type II receptor. Subsequently, the type I receptor is recruited into the complex to form a heterologous tetramer. After binding to the receptor, TGF-β mediates the phosphorylation of Smad2 and Smad3, and forms a complex with Smad4. This complex is translocated into the nucleus and regulates the transcription of target genes, ultimately promoting renal fibrosis. Created in BioRender. Zhou, N. (2026) https://BioRender.com/i7nl7ey (accessed on 29 January 2026).

The TGF-β/Smad axis serves as the principal driver of EMT. TGF-β-mediated Smad2/3 phosphorylation and nuclear translocation activate pro-fibrotic transcriptional programs (Figure 3), leading to persistent ECM accumulation [105]. Recent evidence further shows that Smad3-dependent induction of Foxp2 amplifies EMT, and Foxp2 deletion markedly suppresses matrix production [106]. Additional injury-associated pathways augment this core axis. GPR97 enhances Smad2/3 activation, and its deletion mitigates EMT in DOCA-salt hypertension [107]. The NLRP3 inflammasome promotes IL-1β/IL-18 maturation and forms a positive feedback loop with TGF-β/NF-κB/Smads, while caspase-1/GSDMD-mediated pyroptosis exacerbates the fibrogenic milieu [108].

The Wnt/β-catenin pathway represents the second major mechanism stabilizing EMT and propagating fibrosis. Hypertensive injury suppresses GSK3β, enabling β-catenin accumulation and nuclear entry, thereby activating pro-fibrotic transcriptional programs [109]. It is noteworthy that the functional role of GSK3β remains context-dependent: although its inactivation classically enhances β-catenin signaling and promotes EMT, several studies have reported anti-fibrotic effects of GSK3β inhibition under specific experimental conditions or cell types, potentially through β-catenin-independent regulation of autophagy, inflammation, or lysosomal pathways. These findings highlight mechanistic complexity but do not alter the central pro-fibrotic role of Wnt/β-catenin in EMT [110].

Metabolic and oxidative factors further modulate EMT severity and reversibility. Moderate aerobic exercise elevates irisin and suppresses EMT in spontaneously hypertensive rats, whereas high-intensity exercise lacks such protection [111]. Likewise, negative air ions (NAIs) attenuate nicotine-exacerbated hypertensive renal fibrosis by reducing oxidative stress and inhibiting TGF-β/Smad signaling [112].

Collectively, EMT serves as a pivotal mechanistic bridge linking hypertensive injury to progressive renal fibrosis. The process is coordinated through two major signaling hubs—TGF-β/Smads and Wnt/β-catenin—and is further shaped by GPR97, NLRP3, and metabolic stress, offering multiple promising therapeutic targets for intervention.

### 3.4. Regulatory Mechanisms

#### 3.4.1. Epigenetic Regulation

Epigenetic regulation comprises heritable and reversible modifications in gene expression that occur without changes to the DNA sequence [113]. These regulatory mechanisms—including DNA methylation, histone modifications, and non-coding RNAs—shape chromatin accessibility and transcriptional activity across renal cell types. In hypertension-induced renal injury, epigenetic alterations function as upstream modulators that integrate hemodynamic stress, hormonal factors, and inflammatory signaling into long-term transcriptional reprogramming.

Histone methylation plays a pivotal role in renal transcriptional control. EZH2-mediated H3K27me3 modification has been found to play a key role in maintaining normal podocyte function. Knockdown of EZH2 reduces the level of H3K27me3, which increases susceptibility to glomerular disease in mice, and there may be a similar mechanism that affects podocyte and renal function in hypertension-induced renal injury [114]. Hypertension-related stimuli also influence H3K4 methylation dynamics. Activation of TGR5 modulates H3K4 methylation and represses ENaC expression, thereby attenuating salt-sensitive blood pressure elevation [115]. In kidney development, ASH2L-mediated H3K4me3 is essential for nephron progenitor maintenance, and loss of ASH2L disrupts nephron formation [116].

DNA methylation represents a stable epigenetic mark that establishes long-term transcriptional repression [117]. Hypertensive individuals exhibit increased CpG hypermethylation at key regulatory loci. Hypermethylation of the renin promoter negatively correlates with renin expression in hypertensive patients, suggesting epigenetic suppression of intrarenal RAAS activity [118]. Similarly, KCNK3 promoter hypermethylation reduces expression of a potassium channel essential for vascular tone regulation, contributing to vascular remodeling and hypertension [119]. These findings position DNA methylation as a mechanistic bridge linking vascular dysfunction with downstream renal microvascular injury.

Histone acetylation typically enhances transcriptional activity by loosening chromatin structure [120]. In hypertension-induced renal injury, increased histone deacetylase (HDAC) activity leads to reduced acetylation, repression of protective genes, and enhanced fibrotic signaling [121]. HDAC inhibitors, including Vorinostat and Valproic acid, restore acetylation levels and attenuate renal injury [118]. Acetylation–methylation interactions further modulate injury responses, positioning histone acetylation as a key determinant of whether renal cells adopt reparative or profibrotic phenotypes.

Non-coding RNAs (ncRNAs) include microRNAs (miRNAs) and long non-coding RNAs (lncRNAs), which play important roles in the regulation of gene expression [122]. miRNAs inhibit the translation of mRNAs or promote their degradation by binding to their 3′ untranslated regions (3′UTR). For example, miR-21 is upregulated in hypertension-induced renal injury by targeting PTEN and inhibiting its expression, which in turn activates the PI3K-Akt signaling pathway and promotes cell proliferation and fibrosis. lncRNAs can regulate gene expression through a variety of mechanisms, such as chromatin remodeling, transcriptional regulation, and so on [123]. For example, lncRNA NEAT1 is upregulated in hypertension-induced renal injury and promotes inflammatory and kidney injury by activating the NLRP3 inflammasome. Renal injury can be attenuated by inhibiting specific miRNAs or lncRNAs. For example, the use of miR-21 inhibitors or NEAT1 antisense oligonucleotides (ASOs) reduces their expression levels and attenuates renal fibrosis and inflammatory responses [124]. A recent study observed the conserved lncRNA metastasis-associated lung adenocarcinoma transcript 1 (Malat1) to be elevated in kidney injury, while pharmacological intervention of Malat1 initiated protection against fibrosis. As such, targeting MALAT1 may provide novel strategies to reduce kidney fibrosis [125].

#### 3.4.2. The Gut–Kidney Axis

The gut–kidney axis represents a key regulatory mechanism, integrating metabolic, immune and barrier pathways to dynamically modulate blood pressure and renal homeostasis. Under physiological conditions, microbiota-derived metabolites, including bile acids, short-chain fatty acids (SCFAs), neurotransmitters, and uremic solutes, support renal function, while the kidneys reciprocally regulate intestinal environment by clearing gut-derived toxins and producing hormones [126]. Hypertension and CKD disrupt this regulatory equilibrium, leading to dysbiosis, impaired toxin clearance, increased intestinal permeability, and bacterial translocation, which amplify systemic inflammation and renal injury, reflecting a transition from physiological compensation to pathological decompensation [127].

Within this perturbed environment, microbiota-derived metabolites become major effectors of disease progression. TMAO, markedly elevated in CKD, promotes disease progression by altering lactate production in intrinsic renal cells and inducing macrophage M2 polarization via histone H4K12 lactylation [128]. Sex hormones further shape the gut–kidney axis: androgens elevate blood pressure while reshaping the microbiota and increasing TMA/TMAO levels, contributing to sex-specific differences in hypertension [129].

Barrier–immune crosstalk forms a second amplification loop. TLR4, a key mediator in hypertension, also regulates intestinal permeability and microbial stability. In Ang II-induced hypertension, TLR4 mutation alleviates dysbiosis, prevents hyperpermeability and bacterial translocation, and reduces renal inflammation and dysfunction [130].

The gut–kidney axis extends further to epigenetic–metabolic regulation. In high-fat diet-induced hypertension, depletion of butyrate-producing bacteria leads to reduced circulating and renal butyrate levels, decreased H3K9 butyrylation, and downregulation of MAS1 expression, highlighting an epigenetic mechanism through which microbial metabolites influence blood pressure and renal injury [131]. A causal link is supported by genetic evidence: mice lacking SCFA receptors (GPR41/GPR43) exhibit increased susceptibility to hypertension, suggesting that restoring SCFA–receptor signaling may represent a novel therapeutic strategy [132].

Together, these findings indicate that the gut–kidney axis functions as a multilayered system integrating microbial metabolism, barrier function, immune signaling, and epigenetic regulation, thus offering multiple potential intervention points for treating hypertension-induced renal injury.

#### 3.4.3. Autophagy Dysfunction and Renal Aging

Autophagy represents a key regulatory mechanism maintaining renal cellular homeostasis by degrading damaged organelles, misfolded proteins, and other macromolecular substrates via lysosomal pathways [133]. Under hypertensive conditions, autophagy exerts protective functions by suppressing excessive activation of the NLRP3 inflammasome and limiting the production of pro-inflammatory cytokines such as IL-1β. Because aberrant NLRP3 activation is central to the pro-inflammatory and pro-fibrotic responses in hypertension-induced renal injury, autophagy serves as an essential braking mechanism [134].

However, hypertension-related signals can impair autophagic flux, weakening this protective capacity. Spironolactone has been shown to alleviate hypertension-induced renal fibrosis by enhancing autophagy and inhibiting NLRP3 activation, highlighting autophagy impairment as a mechanistic link between RAAS overactivation and renal injury [135]. In addition, upregulation of specific miRNAs during hypertension suppresses the AMPK–ULK1 pathway, thereby inhibiting autophagy initiation and promoting apoptosis. This leads to insufficient clearance of damaging intracellular substances, further aggravating cellular injury and inflammatory responses [136].

Autophagy dysfunction is also tightly linked to renal aging, a major susceptibility factor for hypertension-induced renal injury. Cellular senescence refers to a state of permanent cell cycle arrest characterized by heterogeneity and complexity. The accumulation of senescent cells in tissues is associated with age-related diseases, including CKD. In the kidney, senescence manifests as tubular atrophy, glomerulosclerosis, and progressive decline in renal function [137].

Senescent cells are distinguished by a unique secretory phenotype known as the senescence-associated secretory phenotype (SASP). The SASP comprises a variety of secreted proteins, cytokines, growth factors, chemokines, and ECM-modifying enzymes [138]. Cellular senescence in both mice and humans, as well as various types of fibrotic diseases, is characterized by iron accumulation. Studies have shown that vascular and hemolytic injury effectively trigger iron accumulation, which in turn induces senescence and promotes fibrosis [139].

In renal proximal tubular cells, oxidative stress activates NF-κB, leading to overexpression of downstream SASP, induction of cellular senescence and upregulation of TGF-β1 and α-SMA, thereby promoting fibrosis [102]. mTOR is a key regulator of SASP secretion. During cellular senescence, autophagosomes and mTOR accumulate within a specialized compartment known as the TOR-autophagy spatial coupling compartment (TASCC). TASCC is associated with the secretion of factors such as TGF-β and CCN2, which play critical roles in the development of renal fibrosis following renal injury [140].

Interestingly, the anti-aging protein Klotho is predominantly produced in the kidney and progressively declines with age [141], which partly due to DNA methylation–mediated transcriptional repression [142]. Klotho deficiency activates the Wnt5a–RhoA pathway, inducing renal vasoconstriction and reducing blood flow, which heightens vulnerability to hypertensive stress. Moreover, conditional deletion of KDM6A in renal tubular cells leads to salt-sensitive hypertension accompanied by reduced Klotho expression and increased aging markers, whereas exogenous Klotho supplementation can rescue this phenotype [143].

Collectively, autophagy impairment diminishes renal resilience to hypertensive injury by weakening anti-inflammatory responses, accelerating cellular aging, and promoting a self-reinforcing cycle of stress accumulation and fibrosis. As a modifiable regulatory mechanism, autophagy represents a promising therapeutic target in hypertension-induced renal damage.

In summary, hypertension-induced renal injury arises from a highly interconnected molecular network rather than a single linear pathway. Hemodynamic stress–induced endothelial dysfunction and microvascular injury act as early initiating events, setting the stage for renal hypoxia, metabolic disturbance, and maladaptive cellular responses. These upstream insults activate multiple amplifying mechanisms, including RAAS overactivation, oxidative stress, immune and inflammatory cascades, and sympathetic nervous system hyperactivity, which engage in extensive cross-talk and self-reinforcing feedback loops across vascular, glomerular, tubular, and interstitial compartments.

Sustained activation of these pathways ultimately converges on common effector processes, most notably EMT, fibroblast activation, and progressive extracellular matrix accumulation, driving irreversible tubulointerstitial fibrosis and functional decline. Importantly, disease susceptibility and trajectory are further shaped by regulatory layers such as epigenetic modification, gut–kidney axis dysregulation, autophagy impairment, and renal aging, which integrate environmental, metabolic, and cellular stress signals.

Collectively, these findings underscore that hypertension-induced renal injury represents a multi-level and dynamic pathogenic network. A comprehensive understanding of these molecular mechanisms not only clarifies the transition from functional disturbance to structural damage but also provides a mechanistic foundation for the development of earlier diagnostic strategies and targeted, disease-modifying therapies discussed in subsequent sections.

## 4. Diagnosis and Monitoring of Hypertension-Induced Renal Injury

**Figure 4:**
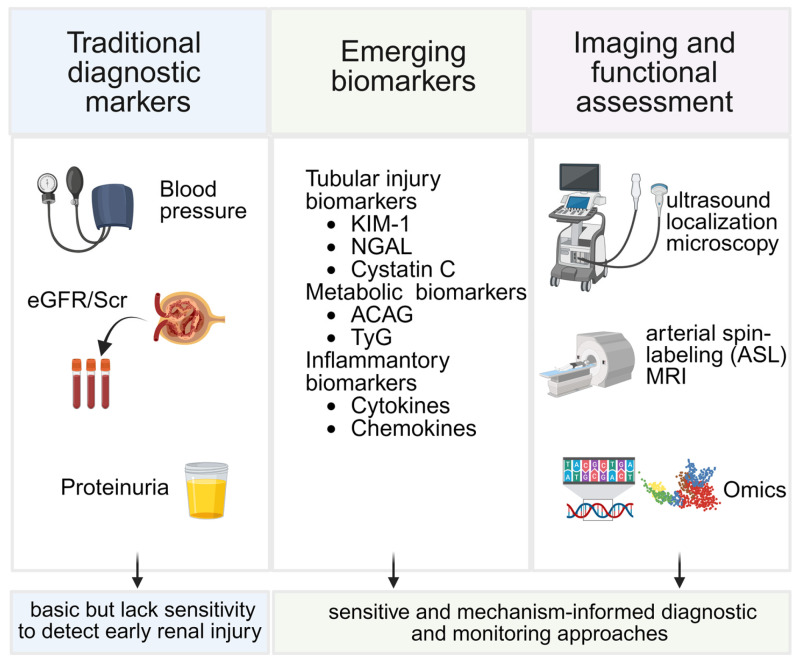
Diagnostic and monitoring strategies for hypertension-induced renal injury. Conventional clinical parameters, including blood pressure, eGFR and albuminuria, provide baseline assessment but have limited sensitivity for early disease. Emerging biomarkers and advanced imaging modalities complement traditional measures, enabling earlier detection, risk stratification and dynamic monitoring of disease progression and therapeutic response. Created in BioRender. Zhang, C. (2026) https://BioRender.com/41wlsc6 (accessed on 23 January 2026).

Hypertension-induced renal injury is often characterized by insidious onset and prolonged subclinical progression, posing substantial challenges for timely diagnosis and risk stratification. Traditional clinical metrics, such as blood pressure levels, serum creatinine, estimated glomerular filtration rate (eGFR), and albuminuria, remain indispensable in routine practice but primarily reflect relatively advanced structural or functional impairment. Increasing evidence indicates that early hypertensive renal injury involves subtle tubular dysfunction, microvascular damage, metabolic disturbances, and inflammatory activation that may not be captured by conventional indicators. Consequently, there is a growing need for more sensitive, mechanism-informed diagnostic and monitoring approaches to enable earlier detection, improved prognostic assessment, and more precise clinical intervention. Based on this, this chapter summarizes the widely used diagnostic markers in clinical practice as well as the emerging biomarkers and imaging methods with diagnostic potent (Figure 4).

### 4.1. Limitations of Traditional Diagnostic Metrics

Hypertension-induced renal injury often progresses silently, and early renal damage may remain undetected using traditional clinical parameters. According to diagnostic criteria, hypertension is defined as a systolic blood pressure ≥140 mmHg or a diastolic blood pressure ≥90 mmHg after repeated measurements [144]. Proteinuria or decline in eGFR typically emerges 5–10 years after the onset of hypertension and reflects relatively advanced disease. Staging based on eGFR (G1–G5) is clinically useful, yet it does not capture microvascular injury or subtle tubular dysfunction during the early phase of hypertensive renal injury.

Proteinuria, commonly assessed using the urinary albumin-to-creatinine ratio (UACR), remains a key diagnostic indicator. Values >150 mg/day or UACR >30 mg/g indicate overt proteinuria, whereas 30–300 mg/g reflects microalbuminuria and >300 mg/g indicates macroalbuminuria [145]. However, albuminuria tends to rise only after significant glomerular barrier disruption or proximal tubular handling abnormalities, meaning that substantial early injury may remain concealed.

Traditional renal function assessment based on serum creatinine and eGFR is limited by biological variability—including muscle mass, age, sex, and hemodynamic factors—and lacks sensitivity to detect early tubular or interstitial pathology [146,147]. As a result, relying exclusively on Scr/eGFR or UACR may delay intervention until irreversible changes such as glomerulosclerosis or tubulointerstitial fibrosis have developed.

Taken together, traditional markers provide important but incomplete information, and they often fail to detect the earliest phases of hypertension-induced renal injury, highlighting the clinical need for more sensitive and mechanism-oriented biomarkers.

### 4.2. Tubular Injury Biomarkers

#### 4.2.1. NGAL

Neutrophil gelatinase-associated lipocalin (NGAL) is a 25 kDa protein that is secreted mostly by immune cells such as neutrophils, macrophages, and dendritic cells. Its production is stimulated in response to inflammation. The concentrations of NGAL can be measured in plasma, urine, and biological fluids such as peritoneal effluent. A research shows that NGAL plasma levels positively correlate with systolic blood pressure, whereas they negatively correlate with urinary Na excretion in subjects of the STANISLAS cohort [148]. Furthermore, a recent study found that, perirenal fat (PRF) thickness positively correlated with urinary concentrations of NGAL and MCP-1 both in correlation analysis and multiple linear regression analysis [149]. NGAL is expected to become an early biomarker for detecting renal failure in patients with hypertension. Recent meta-analyses consistently support NGAL as one of the most sensitive early biomarkers for acute and chronic kidney injury [150]. Its rapid rise following tubular injury precedes detectable changes in creatinine, offering a mechanistically relevant signal particularly useful in hypertension-induced renal injury, where subclinical tubulointerstitial damage may occur before albuminuria becomes evident.

However, NGAL is not kidney-specific and may be elevated in systemic inflammation, infection, or heart failure. This reduces its specificity in clinical settings, necessitating its use in conjunction with complementary biomarkers [151].

#### 4.2.2. KIM-1

KIM-1 is a type I transmembrane glycoprotein that is minimally expressed in normal kidneys but becomes rapidly and robustly upregulated in proximal tubular epithelial cells following ischemic, toxic, or inflammatory insults [152,153]. Upon injury, its extracellular domain is cleaved and shed into urine, making urinary KIM-1 (uKIM-1) a sensitive, non-invasive indicator of early proximal tubular epithelial activation and damage [154].

Mechanistic studies demonstrate that KIM-1 functions as a phosphatidylserine receptor that enables tubular epithelial cells to phagocytose apoptotic debris, linking tubular epithelial clearance pathways, innate immune modulation, and maladaptive remodeling when chronically activated [155]. More recent evidence shows that KIM-1 mediates the uptake of hypoxia-derived small extracellular vesicles, thereby amplifying hypoxia-induced tubulointerstitial inflammation and directly connecting microvascular hypoxia, highly relevant in hypertension-induced renal injury, to progressive interstitial injury [153]. Clinically, both plasma and urinary KIM-1 are strongly associated with subsequent eGFR decline, CKD progression, and kidney failure across multiple cohorts; pKIM-1/uKIM-1 improves risk discrimination beyond conventional predictors [156,157]. Meta-analytic data further support that KIM-1 provides moderate-to-high diagnostic accuracy for tubular injury (pooled AUC ≈0.8), reinforcing its role as an early renal injury biomarker [158].

However, KIM-1 levels may vary with urine flow rate and physiological covariates such as age and albuminuria, and assay platforms remain partly non-standardized [154]. While highly informative for tubular injury and fibrogenic processes, establishing validated clinical cut-offs and prospective utility specifically in hypertensive populations remains an important area for future research.

#### 4.2.3. Cystatin C

Cystatin C is a 13 kDa cysteine protease inhibitor produced at a constant rate by all nucleated cells and freely filtered by the glomerulus. Unlike serum creatinine, it is minimally influenced by muscle mass, age, or diet, making it a more reliable endogenous marker for early GFR impairment [159].

Meta-analytic evidence demonstrates that cystatin C outperforms creatinine-based eGFR equations in predicting cardiovascular events, mortality, and early CKD progression [160]. Because hypertension-induced renal injury involves early microvascular dysfunction and mild reductions in filtration capacity, cystatin C may detect subtle declines before Scr rises.

However, cystatin C levels can be influenced by thyroid disorders, corticosteroid therapy, and systemic inflammation, necessitating cautious interpretation in multimorbid patients.

### 4.3. Metabolic and Biochemical Biomarkers

#### 4.3.1. ACAG

Albumin-corrected anion gap (ACAG) has recently gained attention as a biochemical indicator associated with renal dysfunction in hypertensive patients. A clinical study demonstrated that elevated ACAG serves as an independent risk factor for reduced eGFR (<60 mL/min/1.73 m^2^) and albuminuria (ACR ≥30 mg/g) [161]. Because metabolic acidosis contributes to ammoniagenesis, tubular stress, activation of RAAS, and propagation of interstitial fibrosis, ACAG may reflect early acid–base deviations that precede structural renal injury.

In patients with CKD or early hypertension-induced renal injury, acid–base imbalance often develops before measurable changes in eGFR. ACAG (mmol/L) = [4.4 *−* Albumin (g/dL)] *×* 2.5 + AG may therefore capture subclinical metabolic burden more accurately than AG alone [161]. Recent cohort analyses further show that elevated ACAG is associated with all-cause mortality and adverse renal outcomes in hospitalized populations. These findings support its emerging role as a biochemical “red flag” marker in high-risk hypertensive individuals [162].

Despite these strengths, the diagnostic performance of ACAG remains constrained by methodological issues, including variability in albumin assays, the influence of volume status, and lack of standardized clinical cut-offs. Large-scale prospective studies in hypertensive cohorts are still lacking.

#### 4.3.2. TyG

The triglyceride–glucose (TyG) index is derived from fasting triglyceride and glucose levels. Several meta-analyses indicate that higher TyG values are associated with increased risks of CKD, hypertension, and cardiovascular events [163]. Emerging evidence links TyG to the broader concept of the cardiovascular–kidney–metabolic (CKM) syndrome, underscoring the metabolic–renal–vascular interplay central to hypertension-induced renal injury.

The TyG index also correlates with the triglyceride–glucose–body mass index (TyG-BMI) and markers of systemic insulin resistance, providing additional utility in risk stratification) [164]. Although convenient and inexpensive, TyG is influenced by dietary patterns, acute metabolic shifts, and comorbid diabetes, which may limit its specificity. Nevertheless, TyG represents a promising metabolic biomarker for early identification of patients at high risk for hypertension-induced renal injury.

### 4.4. Omics and Imaging

With the rapid advancement of omics technologies, early diagnostic strategies for hypertension-induced renal injury are increasingly extending beyond conventional functional markers toward molecular-level characterization. Urinary proteomic and metabolomic studies have identified multiple molecular signatures that change prior to overt declines in renal function, providing novel tools for the early prediction of chronic kidney disease progression. Several studies have reported that urinary metabolites, including betaine, choline, and citrate, are significantly associated with future renal function deterioration [165,166]. These metabolic alterations reflect processes such as metabolic reprogramming, mitochondrial stress, and early tubular injury, all of which are key pathological features of hypertension-induced renal injury.

Advanced renal imaging, including ultrasound localization microscopy (ULM), arterial spin-labeling (ASL) MRI, and microvascular perfusion mapping, provides non-invasive visualization of renal microcirculation, complementing biochemical and functional markers [167]. Imaging modalities are particularly beneficial for detecting microvascular rarefaction—a hallmark of hypertension-induced renal injury.

In summary, the diagnosis and monitoring of hypertension-induced renal injury are evolving from reliance on isolated functional parameters toward integrated, multidimensional assessment strategies. Tubular injury biomarkers, metabolic and biochemical indices, omics-based molecular signatures, and advanced imaging techniques each capture distinct but complementary aspects of renal pathophysiology. When considered collectively, these approaches provide a more nuanced evaluation of early renal vulnerability, disease progression, and prognostic risk. Such a mechanism-oriented diagnostic framework not only facilitates earlier detection and refined risk stratification but also lays the foundation for personalized management and timely therapeutic intervention in hypertension-induced renal injury.

## 5. Therapeutic Perspectives of Hypertension-Induced Renal Injury

Hypertension-induced renal injury represents a major contributor to the progression of chronic kidney disease and is closely associated with increased cardiovascular morbidity and mortality. Given its multifactorial pathogenesis, effective management of hypertension-induced renal injury requires an integrated therapeutic strategy that extends beyond simple blood pressure reduction. While adequate blood pressure control remains the cornerstone of treatment, accumulating evidence indicates that neurohumoral dysregulation, metabolic disturbances, inflammation, fibrosis, and maladaptive cellular responses all critically contribute to renal structural damage and functional decline.

Accordingly, this chapter provides a comprehensive overview of current and emerging therapeutic approaches for hypertension-induced renal injury. Established antihypertensive strategies are first discussed as the therapeutic foundation, followed by disease-modifying interventions that confer renoprotective effects beyond blood pressure control, including modulation of sympathetic overactivation. In addition, therapies targeting inflammatory and fibrotic pathways, as well as emerging molecular, cellular, and regenerative strategies, are summarized (Table 1). Together, these approaches highlight the need for individualized and mechanism-based treatment paradigms aimed at slowing disease progression and improving long-term renal and cardiovascular outcomes.

### 5.1. Blood Pressure Control as the Therapeutic Foundation

Effective blood pressure control remains the cornerstone of therapy for hypertension-induced renal injury. Sustained elevation of systemic BP leads to increased intraglomerular pressure, endothelial dysfunction, and progressive structural damage within the kidney. Therefore, antihypertensive therapy constitutes the fundamental therapeutic intervention, upon which additional renoprotective strategies are built.

#### 5.1.1. RAAS Inhibitors

The renin–angiotensin–aldosterone system (RAAS) plays a central role in the initiation and progression of hypertension. Beyond blood pressure reduction, RAAS-targeted therapies aim to attenuate renal inflammation, fibrosis, and progressive structural injury, thereby slowing the development of hypertension-induced renal damage [193]. On this basis, precision anti-inflammatory and anti-fibrotic interventions directed at downstream RAAS signaling pathways are also being actively explored. Recent studies have shown that the deacetylase Sirtuin 6 (Sirt6) protects against renal fibrosis by epigenetically suppressing β-catenin target gene expression [194]. Further evidence indicates that Sirt6 ameliorates renal injury by inhibiting RAAS signaling, which functions downstream of the Wnt/β-catenin pathway. These findings highlight the therapeutic potential of precision strategies targeting the RAAS-related signaling network [195,196].

Although angiotensin-converting enzyme inhibitors (ACEIs) and angiotensin II receptor blockers (ARBs) remain cornerstone therapies for hypertension-related renal injury [168], long-term RAAS blockade is frequently complicated by aldosterone escape, which limits sustained therapeutic efficacy [169]. Meanwhile, it should be emphasized that the use of RAAS inhibitors is subject to safety limitations in specific populations. ACEIs and ARBs must be discontinued during pregnancy due to teratogenic risks, such as fetal calvarial hypoplasia and renal dysgenesis. ACEIs should be stopped and switched to an alternative antihypertensive therapy upon confirmation of pregnancy, whereas ARBs should be discontinued prior to conception [197]. These limitations have driven the development of novel RAAS-targeted strategies. For example, certain novel drugs exert renoprotective effects while simultaneously producing synergistic benefits through modulation of the RAAS signaling pathway. Dapagliflozin has been shown to downregulate the protein expression of ACE and AT1R while upregulating AT2R expression, thereby suppressing RAAS-related pro-inflammatory signaling and attenuating renal inflammation and structural injury. These findings suggest that some non-traditional RAAS-targeting drugs may also contribute to renal protection through indirect regulation of this system [193].

Angiotensinogen, the sole precursor of all angiotensin peptides, represents an upstream and mechanistically attractive therapeutic target. Zilebesiran, a long-acting RNA interference-based therapeutic, suppresses hepatic angiotensinogen synthesis. Clinical studies demonstrate that a single dose of zilebesiran produces sustained blood pressure reductions lasting up to six months in hypertensive patients not receiving antihypertensive medications [170]. Consistently, the KARDIA-2 randomized clinical trial showed that, in patients with uncontrolled hypertension despite treatment with indapamide, amlodipine, or olmesartan, the addition of single-dose zilebesiran resulted in significant reductions in systolic blood pressure at three months, with a low incidence of serious adverse events [171].

Aldosterone dysregulation is another key pathogenic feature in hypertension. Inhibition of aldosterone synthesis represents the most direct strategy for addressing hyperaldosteronism. Baxdrostat, a selective aldosterone synthase (CYP11B2) inhibitor, reduces aldosterone production by blocking its biosynthetic pathway and thereby lowering blood pressure [172]. In a phase 3, multicenter, double-blind, randomized, placebo-controlled trial, the addition of baxdrostat to background therapy significantly reduced seated systolic blood pressure at 12 weeks compared with placebo [198]. However, findings from the phase 2 HALO trial indicated that baxdrostat at doses of 0.5 mg, 1 mg, or 2 mg daily did not significantly reduce blood pressure in patients with uncontrolled hypertension, underscoring variability in clinical responsiveness [199].

More recently, angiotensin receptor–neprilysin inhibitors (ARNIs) have emerged as an additional therapeutic option for hypertension-associated renal injury. By simultaneously inhibiting the angiotensin II receptor and neprilysin, ARNIs modulate the natriuretic peptide system, lower blood pressure, and exert diuretic and vasodilatory effects, thereby improving both cardiac and renal function.

Collectively, emerging RAAS-targeted therapies extend intervention from downstream receptor blockade to upstream ligand suppression and aldosterone synthesis inhibition, offering a more comprehensive strategy for mitigating hypertension-induced renal injury.

#### 5.1.2. Calcium Channel Blockers (CCBs)

CCB inhibits vascular smooth muscle contraction and reduces peripheral vascular resistance by blocking calcium channels, effectively lowering blood pressure. For patients who cannot tolerate RAAS blockers, CCB is a good alternative drug. Studies have shown that the combined use of CCB and RAAS blockers can enhance the anti-hypertensive effect, further reduce proteinuria and delay the deterioration of renal function. In addition, CCB has anti-atherosclerotic effects, which is beneficial to patients with co-morbid cardiovascular disease [173].

The discovery of dual and triple-channel calcium channel blockers represents an advancement beyond traditional L-type CCBs. Cilnidipine, an L/N-type blocker, exerts its effect by modulating sympathetic outflow, since the N-type channels are concentrated on sympathetic nerve terminals. This dual action effectively mitigates sympathetic-mediated hypertension [174]. Additionally, it can protect podocytes by reducing oxidative stress via RAS suppression. In contrast, benidipine (an L/N/T-type blocker) exerts a broader action. The blockade of T-type channels, which are abundant in renal afferent and efferent arterioles, underpins its dual antihypertensive and cardiorenal protective properties [175]. These observations highlight that not all CCBs exert equivalent renoprotective effects, and drug selection may influence renal outcomes beyond BP control alone.

#### 5.1.3. Adjunctive Antihypertensive Therapies

Diuretics and β-blockers are primarily employed as supportive antihypertensive agents in the management of hypertension-induced renal injury, particularly in selected clinical scenarios. Rather than serving as disease-modifying therapies, these agents contribute to blood pressure control and symptom management when first-line strategies alone are insufficient.

Diuretics, including thiazide and loop diuretics, reduce blood pressure by promoting renal sodium excretion and decreasing circulating blood volume. They are especially useful in patients with volume overload or resistant hypertension and are frequently used in combination with other antihypertensive agents to enhance overall efficacy. However, conventional diuretics provide limited evidence for direct renoprotective effects, and their use requires careful monitoring due to the risk of electrolyte disturbances, such as hypokalaemia and hyponatraemia. In patients with impaired renal function, the antihypertensive efficacy of thiazide diuretics is attenuated, and loop diuretics are generally preferred [176].

β-blockers lower blood pressure mainly through inhibition of sympathetic nervous system activity, resulting in reduced heart rate and cardiac output. Although β-blockers are not routinely recommended as first-line agents for isolated hypertension-induced renal injury, they remain clinically relevant in patients with coexisting cardiovascular diseases, including coronary artery disease and heart failure [177]. Carvedilol, a non-selective β-blocker with additional α_1_-adrenergic blocking properties, exhibits antioxidant and antiproliferative effects and is widely used in the treatment of hypertension and heart failure [178]. Arotinolol, a combined α/β-adrenergic blocker, effectively lowers blood pressure without causing significant disturbances in glucose or lipid metabolism, offering potential advantages in patients with metabolic risk factors [179]. More detailed discussion of therapeutic strategies targeting sympathetic overactivation is provided in Section 5.3.

While adequate BP control is indispensable for slowing renal damage, accumulating evidence indicates that BP-lowering alone is insufficient to fully halt the progression of hypertension-induced renal injury. This recognition has driven increasing interest in therapeutic strategies that confer blood pressure-independent renoprotective effects, which are discussed in the following section.

### 5.2. Disease-Modifying Therapies Beyond Blood Pressure Control

Although strict blood pressure control remains the cornerstone of therapy for hypertension-induced renal injury, accumulating evidence indicates that several pharmacological interventions confer renoprotective benefits that extend beyond their antihypertensive effects. These blood pressure-independent mechanisms represent a paradigm shift from blood pressure–centric management toward disease-modifying strategies that directly target renal injury pathways.

#### 5.2.1. SGLT2 Inhibitors

Sodium–glucose cotransporter 2 (SGLT2) inhibitors were initially developed as glucose-lowering agents but are now widely recognized for their robust cardiorenal protective effects. SGLT2 is predominantly expressed in the proximal renal tubules and plays a critical role in glucose and sodium reabsorption. By inhibiting SGLT2, these agents promote glycosuria and natriuresis, leading to modest blood pressure reduction while restoring tubuloglomerular feedback and reducing intraglomerular hypertension [200].

Post hoc analyses of the CREDENCE and CANVAS trials demonstrated that canagliflozin significantly reduced the need for intensive diuretic therapy and slowed the progression of renal disease [180]. Importantly, subsequent studies confirmed that the renoprotective effects of SGLT2 inhibitors are largely independent of baseline estimated glomerular filtration rate (eGFR) or urinary albumin excretion, with consistent benefits observed even in patients with stage 4 chronic kidney disease or microalbuminuria [181]. These findings support the concept that SGLT2 inhibitors function as disease-modifying therapies rather than conventional diuretics.

#### 5.2.2. nsMRA

Mineralocorticoid receptor (MR) overactivation-mediated inflammation and fibrosis are central to the pathogenesis of cardiorenal diseases [201]. Finerenone, a novel non-steroidal mineralocorticoid receptor antagonist (nsMRA), exerts its therapeutic effects by selectively blocking MR overactivation and thereby suppressing downstream inflammatory, immune, and pro-fibrotic signaling pathways [182]. Although finerenone may exert modest blood pressure-lowering effects, its renoprotective actions are largely independent of blood pressure reduction and are primarily mediated through attenuation of renal inflammation and fibrosis.

In contrast to SGLT2 inhibitors, which predominantly target metabolic and hemodynamic pathways and lack direct anti-inflammatory or anti-fibrotic activity, finerenone provides complementary mechanistic benefits. Clinical evidence indicates that combination therapy with finerenone and SGLT2 inhibitors results in a more pronounced reduction in urinary albumin-to-creatinine ratio (UACR) in patients with chronic kidney disease and type 2 diabetes, reflecting their distinct yet synergistic modes of action [202].

Mechanistically, finerenone has been shown to reduce the expression of pro-inflammatory cytokines and to attenuate perirenal macrophage infiltration, thereby mitigating renal immune injury [183]. Furthermore, experimental studies in chronic kidney disease models demonstrate that finerenone ameliorates vascular endothelial dysfunction through activation of the PI3K/Akt pathway and upregulation of endothelial nitric oxide synthase (eNOS), providing additional vasculoprotective effects beyond blood pressure control.

#### 5.2.3. Endothelin Receptor Antagonists

Endothelin-1 (ET-1) is a potent vasoconstrictor peptide produced by vascular endothelial cells and plays a critical role in vascular dysfunction, inflammation, and fibrosis [203]. Endothelin receptor antagonists (ERAs) have therefore attracted considerable interest as therapeutic agents for hypertension and renal disease. Aprocitentan, a once-daily oral dual endothelin receptor antagonist, received its first U.S. approval in March 2024 as an add-on therapy for hypertension, building upon prior clinical investigations of ERAs in this field [184]. However, dose optimization remains essential, as higher doses are associated with a dose-dependent risk of fluid retention [185]. In addition, other ERAs, including sparsentan and zibotentan, are currently under evaluation for their therapeutic potential in hypertension and various nephropathies [186].

Collectively, these therapies exemplify a shift from blood pressure-centric treatment toward mechanism-based interventions that directly target inflammation, fibrosis, and vascular dysfunction in hypertension-induced renal injury.

### 5.3. Modulation of Sympathetic Overactivation

Sympathetic nervous system (SNS) overactivation is a key pathophysiological feature of hypertension-induced renal injury and plays a central role in sodium retention, renin release, renal vasoconstriction, and progressive renal damage. Persistent sympathetic hyperactivity not only contributes to blood pressure elevation but also directly exacerbates renal inflammation, oxidative stress, and microvascular dysfunction, thereby accelerating cardiorenal disease progression.

#### 5.3.1. Pharmacological Modulation of Sympathetic Activity

β-blockers reduce blood pressure primarily through inhibition of sympathetic signaling, leading to decreased heart rate and cardiac output. Beyond their hemodynamic effects, selected β-blockers may partially attenuate sympathetic overactivation-associated oxidative stress and vascular dysfunction. However, β-blockers are not universally indicated for hypertension-induced renal injury and are mainly recommended for patients with concomitant cardiovascular diseases, such as coronary artery disease or heart failure. Therefore, their role in this context is best viewed as symptom- and comorbidity-oriented rather than disease-modifying.

#### 5.3.2. RDN

Renal denervation (RDN) has emerged as an interventional strategy targeting renal sympathetic nerves, motivated by the high prevalence of poor medication adherence and drug intolerance in patients with hypertension. By disrupting efferent and afferent renal sympathetic signaling, RDN reduces renal vasoconstriction, sodium reabsorption, and renin secretion, thereby lowering blood pressure and sympathetic tone [187,188]. Currently, RDN is primarily considered for patients with resistant hypertension accompanied by sympathetic overactivation.

Recent clinical and experimental studies suggest that RDN may exert additional renoprotective effects beyond blood pressure reduction. Notably, selective afferent renal denervation has been shown to improve renal function in chronic kidney disease models, indicating a potential role in modulating central sympathetic outflow and renal inflammation. With the approval of multiple RDN devices by regulatory agencies, this approach may open new therapeutic avenues for cardiorenal diseases, although long-term renal outcomes require further validation [204].

#### 5.3.3. GLP-1 Receptor Agonists

Emerging evidence has identified the carotid bodies (CBs) as critical peripheral chemosensors linking metabolic dysfunction to sympathetic overactivation. Glucagon-like peptide-1 receptor (GLP-1R) expression has been detected in CB glomus cells in both rodents and humans [205,206]. Reduced GLP-1R signaling in the CB is associated with enhanced chemoreflex sensitivity and sympathetic hyperactivity in cardiometabolic disease models [207].

Experimental studies demonstrate that GLP-1 receptor agonists (GLP-1RAs) attenuate baseline CB sensory discharge and blunt chemoreflex-induced increases in blood pressure and sympathetic nerve activity. These findings suggest that the GLP-1R–CB pathway represents a novel mechanism through which GLP-1RAs may modulate sympathetic overactivation, complementing their established metabolic, anti-inflammatory, and cardiovascular benefits [189]. This pathway provides a mechanistic link between metabolic regulation and neurohumoral control in hypertension-induced renal injury.

Collectively, therapeutic strategies targeting sympathetic overactivation, from pharmacological modulation to device-based and neuro-metabolic interventions, highlight the SNS as a critical and multifaceted therapeutic axis in hypertension-induced renal injury.

### 5.4. Anti-Inflammatory and Anti-Fibrotic Strategies

Chronic inflammation and progressive fibrosis represent convergent downstream pathways in the development and progression of hypertension-induced renal injury. Beyond hemodynamic stress, sustained inflammatory activation, immune dysregulation, and excessive extracellular matrix deposition collectively drive irreversible structural remodeling and functional decline of the kidney. Accordingly, therapeutic strategies targeting inflammatory and fibrotic processes have attracted increasing attention as important complements to blood pressure control and neurohumoral modulation.

Rather than constituting a single, unified therapeutic category, anti-inflammatory and anti-fibrotic interventions are implemented through diverse approaches with distinct mechanistic bases. These include traditional and emerging interventions, each of which addresses specific components of inflammatory and fibrotic signaling networks.

### 5.5. Emerging Therapies

#### 5.5.1. Molecularly Targeted Approaches

In recent years, molecularly targeted therapies have attracted increasing attention as emerging strategies for the treatment of hypertension-induced renal injury. Gene therapy and gene-editing approaches represent important directions in this field [208]. As discussed in Section 3, multiple key regulatory molecules participate in the development and progression of hypertension-induced renal injury, and targeted modulation of these pathways holds therapeutic potential.

Beyond the mechanisms addressed in earlier sections, additional molecular targets have been identified. For instance, sirtuin 7 (SIRT7) has been shown to attenuate ferroptosis, epithelial–mesenchymal transition (EMT), interstitial fibrosis, and both functional and structural renal damage in hypertensive mouse models by promoting KLF15/Nrf2 signaling. Accordingly, targeting SIRT7-related pathways may represent a promising molecular strategy for the treatment of hypertension and hypertension-induced renal injury [190]. Nevertheless, the clinical applicability and long-term safety of such molecularly targeted interventions require further investigation.

#### 5.5.2. Stem Cell-Based Therapies

Stem cell-based therapies have shown considerable promise for the treatment of hypertension-induced renal injury owing to their self-renewal capacity and multidirectional differentiation potential [209]. Mesenchymal stem cells (MSCs) can exert renoprotective effects by secreting a variety of cytokines and growth factors, thereby inhibiting inflammation, modulating immune responses, and promoting renal cell repair and regeneration. In addition, MSCs are capable of homing to injured renal tissue and differentiating into renal cell types, contributing to the replacement of damaged cells.

Experimental studies and limited clinical investigations have demonstrated that MSC therapy can improve renal function, reduce proteinuria, and alleviate renal pathological injury in models of hypertension-induced renal damage [191]. However, critical issues, including the optimal cell source, transplantation route, dosage, and long-term safety, remain to be clarified before stem cell therapy can be widely applied in clinical practice.

#### 5.5.3. EV-Based Therapies

Extracellular vesicles (EVs) have emerged as promising therapeutic tools in regenerative medicine. EVs can mediate intercellular communication by transporting bioactive molecules, including proteins, lipids, and nucleic acids, thereby influencing inflammation, fibrosis, and tissue repair. However, the therapeutic efficacy of current exogenously administered EV-based strategies for chronic tissue injury remains limited.

Recent studies suggest that in vivo reprogramming of EV secretion through metabolic engineering represents a novel approach for enhancing therapeutic efficacy. This strategy enables coordinated regulation of EV biogenesis and cargo-sorting pathways, thereby improving their potential for treating chronic diseases, including hypertension-associated organ damage [192]. Despite these advances, EV-based therapies remain at an early developmental stage, and substantial challenges related to standardization, delivery, and safety must be addressed.

### 5.6. Lifestyle Management

Lifestyle modification remains a fundamental component in the management of hypertension-induced renal injury. Beyond lowering blood pressure, non-pharmacological interventions may attenuate renal hemodynamic stress, improve metabolic abnormalities, and reduce systemic inflammation, thereby influencing upstream pathogenic drivers [210]. Dietary sodium restriction enhances blood pressure control and potentiates the antiproteinuric effects of RAAS blockade. Lower sodium intake reduces intraglomerular pressure, particularly in salt-sensitive individuals [210]. Weight management and regular physical activity improve insulin resistance, endothelial dysfunction and sympathetic overactivity. In obesity-related hypertension, modest weight loss has been associated with reductions in albuminuria and slower eGFR decline [211]. Smoking can lead to the progression and deterioration of the disease. Therefore, all smokers should be strongly advised to quit smoking [212]. Although lifestyle management alone is insufficient in established disease, it provides an essential foundation that enhances the effectiveness of pharmacological therapy and may contribute to slowing disease progression.

In summary, the management of hypertension-induced renal injury relies on a comprehensive and mechanism-based therapeutic framework. Effective blood pressure control remains the fundamental prerequisite for renal protection, while increasing attention has been directed toward disease-modifying interventions that confer benefits beyond hemodynamic regulation. Modulation of sympathetic overactivation, together with strategies targeting inflammatory and fibrotic pathways, represents important complementary approaches for attenuating renal structural damage and functional decline.

In parallel, emerging therapeutic strategies, including molecularly targeted approaches, stem cell-based therapies, and extracellular vesicle-mediated interventions, have demonstrated promising renoprotective potential in experimental and early clinical studies. However, their translation into routine clinical practice requires further validation with respect to efficacy, safety, and long-term outcomes. Collectively, these considerations underscore the importance of individualized and integrative treatment paradigms aimed at slowing disease progression and improving long-term renal and cardiovascular prognosis in patients with hypertension-induced renal injury.

## 6. Conclusions and Discussion

Hypertension-induced renal injury represents a complex, multi-layered process driven by persistent hemodynamic stress and reinforced by metabolic, inflammatory, and fibrotic amplification loops. Increasing evidence suggests that hypertension-induced renal injury is not merely a passive consequence of elevated blood pressure, but the result of a dynamically evolving pathogenic network.

Looking forward, several key challenges remain. Firstly, the lack of available biomarkers and functional imaging for clinical use to identify high-risk individuals early makes it difficult to provide timely intervention before irreversible fibrosis occurs. Secondly, while emerging therapies such as SGLT2 inhibitors, GLP-1RAs and MRA show promise, optimal sequencing, combination strategies and stage-specific application require prospective validation in hypertension-dominant populations. In addition, translating complex molecular insights into clinically actionable precision medicine frameworks remains an ongoing challenge.

Future research should move beyond single-pathway paradigms toward structured network modulation, integrating mechanistic insights with precision clinical phenotyping. Addressing these challenges may shift the management of hypertension-induced renal injury from reactive blood pressure control to proactive, mechanism-guided disease modification.

## Figures and Tables

**Table 1:** Therapeutic strategies for hypertension-induced renal injury.

Therapeutic Category	Representative Agents/Strategies	Representative Drugs	Renal Protective Effects	References
Blood pressure control	RAAS inhibition	ACEI, ARB, Zilebesiran, Baxdrostat, ARNI	Improved renal hemodynamics, reduced intraglomerular pressure and partial attenuation of inflammation and fibrosis	[168,169,170,171,172]
	Calcium channel blockers	Nifedipine, Cilnidipine, benidipine		[173,174,175]
	Diuretics	Furosemide, Torsemide		[176]
	β-blockers	Metoprolol, Carvedilol, Arotinolol		[177,178,179]
Disease-modifying therapies	SGLT2 inhibitors	Canagliflozin, Empagliflozin	Promote glycosuria and natriuresis, reduce intraglomerular hypertension and restore tubuloglomerular feedback	[180,181]
	nsMRA	finerenone	Suppress inflammatory, immune, and pro-fibrotic signaling pathways	[182,183]
	Endothelin receptor antagonists	Aprocitenta, sparsentan, zibotentan	Attenuate vasoconstriction, inflammation, and fibrosis.	[184,185,186]
Sympathetic inhibition	Renal denervation	-	Potential reduction of renal sympathetic overactivity	[187,188]
	GLP-1 receptor agonists	Liraglutide, exenatide		[189]
Emerging therapies	Molecularly targeted approaches	SIRT7, etc.	Modulate pathogenic signaling, enhance endogenous repair, and attenuate inflammatory and fibrotic responses.	[190]
	Stem cell-based therapies	Mesenchymal stem cells (MSCs)		[191]
	EV-based therapies	Reprogramming extracellular vesicles		[192]

RAAS, The renin–angiotensin–aldosterone system; ACEI, angiotensin-converting enzyme inhibitors; ARB, angiotensin II receptor blockers; SGLT2, Sodium–glucose cotransporter 2; nsMRA, non-steroidal mineralocorticoid receptor antagonist.

## Data Availability

No new data were created or analyzed in this study.
